# Pharmacogenetics and adverse drug reports: Insights from a United Kingdom national pharmacovigilance database

**DOI:** 10.1371/journal.pmed.1004565

**Published:** 2025-03-27

**Authors:** Emma F. Magavern, Maia Megase, Jack Thompson, Gabriel Marengo, Julius Jacobsen, Damian Smedley, Mark J. Caulfield

**Affiliations:** 1 William Harvey Research Institute, Queen Mary University of London, London, United Kingdom; 2 Department of Clinical Toxicology and General Medicine, St Thomas’ Hospital, Guy’s and St Thomas’ NHS Foundation Trust, London, United Kingdom; University of Oxford, UNITED KINGDOM OF GREAT BRITAIN AND NORTHERN IRELAND

## Abstract

**Background:**

Adverse drug reactions (ADRs) harm patients and are costly for healthcare systems. Genetic variation contributes to variability in medication response and prospective knowledge of these variants can decrease risk of ADRs, as shown in the PREPARE trial. Reduction in ADRs would affect only those reactions to drugs contained in well-validated pharmacogene–drug pairs. The scope of ADRs represented by these drugs on a population scale is unclear. The objective of this study was to characterize the pharmacogene–drug-associated ADR reporting landscape from a national regulatory pharmacovigilance dataset to elucidate the scale of potential ADR mitigation by pharmacogenomics (PGx) implementation.

**Methods and findings:**

All publicly available Yellow Card ADR reports to the United Kingdom Medicines and Healthcare Products Regulatory Agency, from 1963 to 2024, were compiled using programmatic data extraction. The ADRs were analysed with descriptive statistics, stratified by PGx status and by associated genes. Prescribing prevalence from the literature was compared with age range matched ADR reports for PGx-associated drugs.

There were 1,345,712 ADR reports, attributed to 2,499 different substances. 115,789 adverse drug reports (9%) were associated with drugs for which ADR risk can be modified based on pharmacogenomic prescribing guidance. Seventy-five percent of these (*n* = 87,339) were due to medicines which interact with only three pharmacokinetic pharmacogenes (*CYP2C19*, *CYP2D6*, *SLCO1B1*). Forty-seven percent of all the PGx mitigatable ADRs identified were attributed to psychiatric medications (*n* = 54,846), followed by 24% attributed to cardiovascular medications (*n* = 28,279). Those experiencing PGx mitigatable ADRs, as compared with non-PGx mitigatable ADRs, were older and the ADRs more often consisted of severe non-fatal reactions. Many PGx-associated psychiatric drug ADRs were overrepresented as compared with prescribing prevalence, but fatal cardiac arrhythmias were uncommon consequences, comprising only 0.4% of these ADRs (*n* = 172 of *n* = 48,315 total ADRs).

Limitations of this data source include under reporting of ADRs and reporting bias. These findings are based on analysis of the Yellow Card dataset described and may not represent all ADRs from a generalised patient population.

**Conclusions:**

Nine percent of all reported ADRs are associated with drugs where a genetic variant can cause heightened risk of an ADR and inform prescribing. A panel of only three pharmacogenes could potentially mitigate three in every four PGx modifiable ADRs. Based on our findings, Psychiatry may be the single highest impact specialty to pilot PGx to reduce ADRs and associated morbidity, mortality and costs.

## Author summary

**Why was this study done?:**

- Side effects to medications (adverse drug reactions (ADRs)) are common and can be dangerous.
- Though some side effects could be avoided by personalising prescribing based on genetic variants (pharmacogenonomics (PGx)) it is not clear what volume of side effects are reported from medications that could be personalised using this approach.
- This study used the United Kingdom national side effect reporting system, called the Yellow Card reports, to estimate the volume of ADRs that could be impacted on with personalised prescribing from the past 60 years.

**What did the researchers do and find?:**

- All publicly available Yellow Card reports to the United Kingdom Medicines and Healthcare Products Regulatory Agency from, 1963 to 2024, were compiled and analysed. Prescribing prevalence from the literature was compared with side effect reports for PGx-associated drugs.
- There were over 1 million reports of side effects to medications, attributed to more than 2000 different substances. Nine percent of these reported side effects were associated with drugs for which side effect risk can be modified using genetic information to guide prescribing.
- Seventy-five percent of these PGx mitigateble side effects were associated with medicines which interact with only three genes that impact how the body processes medication (*CYP2C19*, *CYP2D6*, *SLCO1B1*).
- Forty-seven percent of all the PGx mitigatable side effects identified were attributed to psychiatric medications, followed by 24% attributed to cardiovascular medications. Reported side effect to many PGx-associated psychiatric drugs were overrepresented as compared with how commonly the medications were prescribed.

**What do these findings mean?:**

- Nine percent of all spontaneously reported side effects are associated with drugs where a genetic variant can cause heightened risk of a side effect and inform prescribing.
- Using a panel of only three genes could potentially mitigate three in every four PGx modifiable side effects.
- Based on our findings, Psychiatry may be the single highest impact specialty to pilot pharmacogenomics to reduce side effects.
- This study is based on the reports submitted voluntarily by healthcare practitioners and members of the public to the database and may not represent all ADRs.

## Abstract 3

Emma F Magavern, Maia Megase and colleagues use Yellow Card reports from the past 60 years to estimate the volume of adverse drug reactions that could be targeted by personalised prescribing based on pharmacogenomics.

## Introduction

There is significant variability in interindividual response to medication. Some of this variable response, which contributes to adverse drug reactions (ADRs), is due to interaction between genetic variants and medications [1]. This is often referred to as pharmacogenomics (PGx). Population scale studies have shown that 99.5% of individuals have genetic variants which could lead to an atypical drug response [2].

ADRs are common and costly, impacting heavily on patient quality of life, medication adherence, and even morbidity and mortality [3]. Recent estimates place the cost of ADRs at greater than 2.2 billion pounds per annum in the United Kingdom (UK), and ADRs are estimated to represent 6.5% of all acute hospital admissions [4,5].

As the population in the UK ages, a trend seen in many high income countries, patients are living longer with more medical morbidities, contributing to both appropriate and inappropriate polypharmacy [6–8]. This has led to an increase in number of prescriptions dispensed nationally between the last two census points [9,10]. An estimated 3.8 million people in England alone take eight or more medicines [7]. These population level trends highlight the importance of reducing risk factors for ADRs and have led to a surge in interest in PGx implementation whereby prospective genotyping enables personalised prescribing.

Prospective clinical trial evidence from the U-PGx group (PREPARE trial—Pre-emptive Pharmacogenomic Testing for Preventing ADRs) shows that implementation of a PGx panel can reduce ADRs by 30% [11]. Internationally recognised PGx consortia guidelines, such as those produced by the Dutch Pharmacogenetics Working Group (DPWG) (implemented by the PREPARE trial) or the Clinical Pharmacogenetics Implementation Consortium (CPIC), provide clinically actionable guidance for a subset of drugs with well validated drug–gene relationships [12,13]. The scope of ADRs arising from drugs with PGx guidance in the context of all reported ADRs across a population is currently unclear. None of the regulators, to our knowledge, have reported retrospectively on the percentage and composition of reporting for drugs with PGx indications. We have retrospectively analysed all Yellow Card ADR reports published by the UK Medicines and Healthcare Product Regulatory Agency (MHRA), encompassing reports from 1963 to 2024, to address this gap in knowledge.

The objective of this study was to characterize the pharmacogene–drug-associated ADR reporting landscape from a national regulatory pharmacovigilance dataset to elucidate the scale of potential ADR mitigation by PGx implementation in the UK. Furthermore, we explored a possible association between demographic variables and ADR reports which can be mitigated by implementation of PGx. Such knowledge may impact on choice of initial PGx implementation settings in the UK.

## Methods

### Data source: Yellow Card reports

The UK MHRA established the Yellow Card reporting system in the 1960s following the Thalidomide tragedy, thereby creating a national pharmacovigilance system to ensure medication safety. These reports for ADRs can be submitted by patients themselves, carers, or healthcare practitioners as well as by industry teams. The MHRA makes these reports publicly available on their website via interactive drug analysis profiles (iDAPs) which allow visualization of reports by active ingredient [14]. Information is provided for various parameters such as: age and sex of the patient, the year of the report, the type of reporter, the severity of the reported ADR and the Medical Dictionary for Regulatory Activities terms for the ADRs reported. The MHRA provide guidance on the appropriate interpretation of the strengths and limitations of this data, which can be found on any given iDAP page (for example https://info.mhra.gov.uk/drug-analysis-profiles/dap.html?drug=./UK_EXTERNAL/NONCOMBINED/UK_NON_000408008276.zip&agency=MHRA). Reports are spontaneous and not proven to be related to the linked substance. Reporting of ADRs has consistently increased over time, likely in part due to increased awareness, therefore all analyses are performed with normalised statistics, showing percentage of ADRs by decade, rather than absolute report numbers [15].

### Compilation of a master ADR document

Data was extracted from the MHRA website on May 17, 2024. Files were downloaded using the package rvest 1.0.4 and then compiled into a master doc using the following packages: future 1.33.2, collapse 1.10.6, fst 0.9.8, future.apply 1.11.2, dplyr 1.1.2 and readr 2.1.4. The drug, event and case files supplied as individual csv files on the MHRA website were merged for each ADR report for each drug then combined to make a master file with all publicly available Yellow Card reported ADR information. All programmatic data extraction and data wrangling was performed using R 4.3.0. The code used can be found in the github repository listed here (https://github.com/whri-phenogenomics/yellow_card_pharmacogenomic_project/tree/main).

### Analysis of the Yellow Card reported ADRS

Thirty-nine drugs from the PREPARE trial were included in our list of genetically actionable drugs to estimate volume of reported ADRs that could be modified by using PGx information (S1 Table) [11]. We did not include drugs not licensed in the UK (phenprocoumon) or that were deemed unactionable mid-way through the PREPARE trial (clozapine and oxycodone). These PGx medications were grouped to show the number and percentage of ADR reports associated with PGx actionable drug–gene pairs. We therefore limited our assessment of clinically actionable gene–drug pairs to those with impact on ADRs rather than purely efficacy, with stringent evidence criteria, and prospective clinical trial data demonstrating reduction in ADRs with PGx implementation.

To assess the potential impact of mitigating ADRs with PGx for prescribing in primary care, we utilized work from a longitudinal English study [16]. We applied published data from the Clinical Practice Research Datalink (CPRD) on prescribing prevalence of PGx medications in English primary care to assess the relationship between medication prescribing prevalence and ADR report prevalence from the same drug in the Yellow Card dataset [16]. This was done to look for any medications or genes which were overrepresented in Yellow Card reports with respect to prescribing prevalence. The data on prevalence of prescribing in the representative CPRD cohort over a 20-year period from 1993 to 2012 was used and compared with Yellow Card ADRs reported for patients in the same age range (30 to 99-years old) during that same time period [16]. Percentage of ADR reports from the drug of interest were analysed using all ADR reports for our PGx drug list as the denominator to look only at PGx mitigatable Yellow Card ADRs.

Oestrogen reported ADRs were excluded from our PGx mitigatable list to give the most robust conservative estimate of PGx mitigatable ADRs. Oestrogens were not considered as an inclusion criterion in the PREPARE trial. As only specific ADRs would be mitigatable via pharmacodynamic interaction with the *F5* genetic variant causing factor V Leiden (FVL) hypercoagulability, we opted to exclude them from the list of PGx mitigatable drugs shown above. However, Oestrogens are known to synergistically increase risk of venous thromboembolism (VTE) in patients with FVL leading to PGx mitigatable deep venous thrombosis (DVT) and pulmonary embolism (PE), which can be life threatening complications. We have therefore analysed the number and percentage DVT and PE ADRs for all oestrogens in the Yellow Card reports, as well as the fatality associated with these reports.

Given that the highest speciality for intervention to target mitigatable ADRs appeared to be psychiatry, based on the results of the above analyses, we analysed the ADR reactions to quantify the number, percentage and fatality associated with reported arrythmias for the PGx mitigatable antidepressant and antipsychotic drugs (including citalopram, escitalopram, paroxetine, sertraline, venlafaxine, amitriptyline, clomipramine, doxepin, imipramine, nortriptyline, aripiprazole, haloperidol, pimozide, and zuclopenthixol).

### Statistical methods

The percentage of Yellow Card reports made in association with medications linked with pharmacogenomic indications were quantified by decade. The denominator of all Yellow Card reports per decade was used to show the percentage of reported ADRs that may have been intervened on with PGx testing through the history of Yellow Card reporting and account for dynamic shifts in prescribing that occur naturally over the 60-year period of Yellow Card reporting.

This reporting landscape was then analysed by available demographics such as sex and age to look for differences in reporting across PGx actionable drugs and non PGx actionable drugs and noting missing demographic data. Age at the time of ADR is given by the Yellow Card reports in decade bands (i.e., 20 would encompass ages 20- to 29-years old). Reports were stratified by disease area of indication to examine how these examples of potentially PGx mitigatable ADRs are spread across specific specialty prescribers and organ systems.

The 39-drug list described above is a limited subset of known PGx-associated drugs that have DPWG therapeutic recommendations. As a sensitivity analysis we assessed a more extensive CPIC clinically actionable drug list defined as level A and associated with ADR risk, to give a less conservative estimate of potential scale of PGx mitigatable ADRs from the Yellow Card data set.

This study is reported as per the Strengthening the Reporting of Observational Studies in Epidemiology (STROBE) guidelines (S1 STROBE Checklist).

#### Ethics.

Ethics approval was not required due to the use of public domain data.

## Results

**Fig 1:**
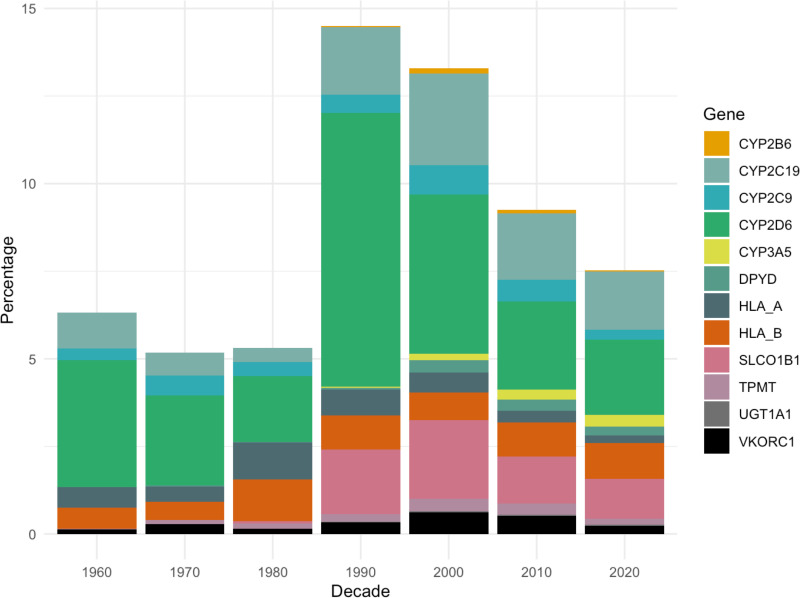
Percentage of ADR reports for PGx drugs and associated genes by decade. The *x*-axis shows decade of ADR report to reflect changes in prescribing trends over time. The total number of ADRs reported in each decade are as follows: 1960 = 21,341, 1970 = 65,318, 1980 = 132,500, 1990 = 181,754, 2000 = 229,721, 2010 = 396,779 and 2020 = 318,299. ADR, adverse drug reaction; PGx, pharmacogenomics.

There were 1,345,712 ADR reports over the span of Yellow Card reporting, from 1963 to 2024, attributed to 2,499 different substances. Of these ADR reports, 5% were fatal (*n* = 61,944) and 65% were serious but non-fatal (*n* = 874,564) according to the Council for International Organizations of Medical Sciences criteria used by the MHRA. From this dataset, 9% (*n* = 115,789) of ADR reports were associated with drugs for which ADR risk can be modified based on DPWG pharmacogenomic prescribing guidance as demonstrated by results of the PREPARE trial. These 115,789 drug ADRs, which may have been partly due to gene–drug interactions, involved 12 genes. 75% (*n* = 87,339) of these ADRs were attributed to medicines which interact with only three pharmacokinetic pharmacogenes (*CYP2C19*, *CYP2D6*, *SLCO1B1*), with an additional 6% (*n* = 7,215) attributed to medications which interact with a fourth pharmacokinetic pharmacogene (*CYP2C9*). Fig 1 shows the percentage of pharmacogene-associated medication ADR reports by decade to correct for changes in prescribing trends over time. The underpinning data is shown in S2 Table. The smallest percentage of PGx mitigatable ADRs were reported in the 1970s, where they represented 5% of all Yellow Card reports. The largest was in the 1990s, during which they encompassed 15% of all Yellow Card reports.

### Pharmacogenes and ADRs

**Fig 2:**
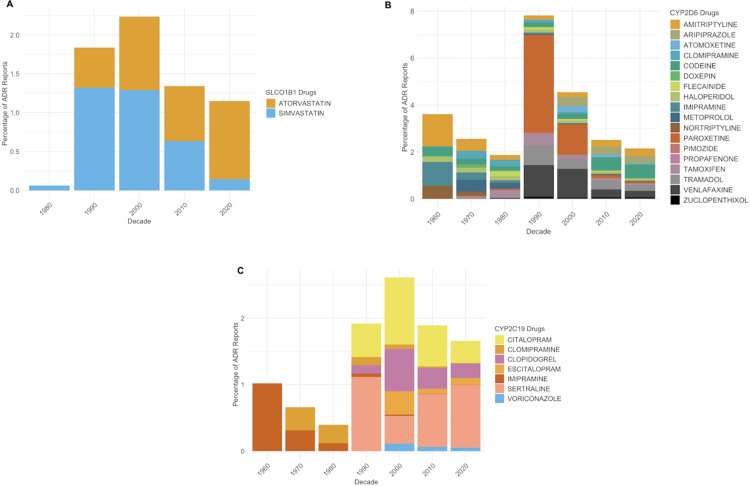
Percentage of adverse drug reactions (ADRs) for pharmacogene-associated medications. **(A)** Percentage of ADRs for *SLCO1B1*-associated medications. **(B)** Percentage of ADRs for *CYP2D6*-associated medications. **(C)** Percentage of ADRs for *CYP2C19*-associated medications. For all graphs, the *x*-axis shows decade of ADR report to reflect changes in prescribing trends over time, while each contributing drug agent associated with *SLCO1B1*, *CYP2D6* and *CYP2C19* is shown with percentage of total ADR reports per decade on the *y*-axis. Statin-associated ADR reports start in the 1980s, which is when they emerged as licensed therapeutic agents. ADR, adverse drug reaction.

Fig 2A–2C show the individual medications contributing to the PGx mitigatable reports over time from the top three pharmacogenes of interest. SLCO1B1 is associated with the use of statins, so trends were clearly interpretable over time, but there has been a shift from simvastatin- to atorvastatin-related ADRs over time (S3 Table). Trends in Yellow Card ADRs from the CYP2D6 and CYP2C19 metabolised medications (many of which are antidepressants, antipsychotics or analgesics) by decade are shown in Fig 2B and 2C (S4 and S5 Tables). This revealed, for example, that the peak in the 1990s in CYP2D6 drug-related reporting was due to paroxetine. With regards to CYP2C19, tricyclic antidepressant-associated reporting has decreased over time, but serotonin reuptake inhibitors- (SSRIs) associated ADR reports have increased, as prescribing practice has shifted to SSRIs (as seen by progressively lower reports of imipramine and clomipramine ADRs and the emergence of sertraline ADRs after licensing in 1990).

### Variables associated with PGx mitigable ADRs

Table 1 shows patient demographics, ADR severity and reporter details for the PGx mitigatable and non PGx mitigable ADRs. Though Yellow Card reports are overall more common in females, there was a higher percentage of males with PGx mitigatable ADRs than non-PGx mitigatable ADRs. Patients who experienced PGx mitigatable ADRs were older and had more severe but non-fatal reactions as compared with patient who experienced a non-PGx mitigatable ADR. PGx mitigatable ADRs were more likely to be reported by patient or carers and more likely to be reported directly to the MHRA rather than reported from industry.

**Table 1:** ADR report numbers and patient demographics stratified by drug PGx status. Sex, age and severity comparison between pharmacogene actionable medicines and non pharmacogene actionable medicines.

	Total ADRs	PGx drug ADRs	Other ADRs
	(*N* = 1,345,712)	(*N* = 115,789)	(*N* = 1,229,923)
**Male (%)**	38% (*n* = 512,241)	40% (*n* = 45,876)	38% (*n* = 466,365)
**Female (%)**	57% (*n* = 769,852)	56% (*n* = 64,903)	57% (*n* = 704,949)
(Missing data %)	5% (*n* = 63,619)	4% (*n* = 5,010)	5% (*n* = 58,609)
**Age (mean** ± **SD)**	46.9 (±21.1 years)	47.1 (±20.5 years)	46.9 (±21.1 years)
(Missing data %)	22% (*n* = 289,118)	16% (*n* = 18,192)	22% (*n* = 270,926)
**Severity**			
**Nonserious (%)**	30% (*n* = 409,188)	27% (*n* = 30,678)	31% (*n* = 378,510)
**Serious (%)**	65% (*n* = 874,564)	69% (*n* = 80,062)	64% (*n* = 794,502)
**Fatal (%)**	5% (*n* = 61,944)	4% (*n* = 5,049)	5% (*n* = 56,895)
(Missing data %)	0% (*n* = 16)	0% (*n* = 0)	0% (*n* = 16)
**HCP reporter (%)**	80% (*n* = 1,082,961)	80% (*n* = 93,082)	80% (*n* = 989,879)
**Patient/Carer (%)**	16% (*n* = 213,597)	17% (*n* = 19,247)	16% (*n* = 194,350)
**Both %**	4% (*n* = 49,154)	3% (3,460)	4% (*n* = 45,694)
**Direct reports (%)**	67% (*n* = 903,325)	75% (*n* = 86,665)	66.4% (*n* = 816,660)
**Indirect reports (%)**	33% (*n* = 442,387)	25% (*n* = 29,124)	33.6% (*n* = 413,263)
(Missing data %)	0% (*n* = 0)	0% (*n* = 0)	0% (*n* = 0)

ADR, adverse drug reaction; PGx, Pharmacogenomic; SD, Standard deviation; HCP, Healthcare professional. Indirect reports come from industry as compared with direct reports to the MHRA. There were no counts of missing data from Direct vs Indirect reporter category.

### Specific drugs and ADRs

**Fig 3:**
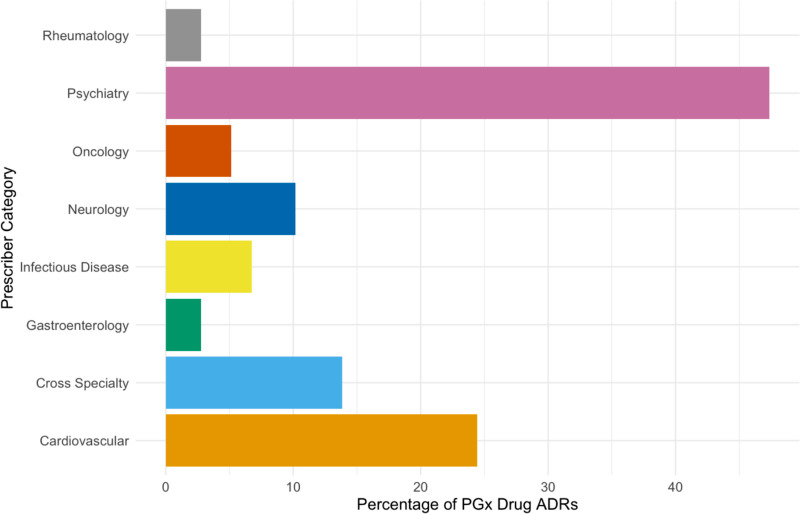
Percentage of PGx mitigatable ADRs by prescriber specialty. The breakdown of prescribing indication by specialty can be found in S1 Table. The following were classified as Rheumatology medications: azathioprine, mercaptopurine. The following were classified as Psychiatry medications: citalopram, escitalopram, paroxetine, sertraline, venlafaxine, amitriptyline, clomipramine, doxepin, imipramine, nortriptyline, carbamazepine, aripiprazole, haloperidol, pimozide, zuclopenthixol, atomoxetine. The following were classified as Oncology medications: capecitabine, 5-fluorouracil, irinotecan, tamoxifen, tegafur, thioguanine, mercaptopurine. The following were classified as Neurology medications: phenytoin, carbamazepine, azathioprine, mercaptopurine. The following were classified as Infectious Disease medications: efavirenz, flucloxacillin, voriconazole. The following were classified as Gastroenterology medications: azathioprine, mercaptopurine. The following were classified as Cross Speciality medications: codeine, tramadol, tacrolimus, azathioprine, mercaptopurine. The following were classified as cardiovascular medications: flecainide, propafenone, metoprolol, atorvastatin, simvastatin, acenocoumarol, clopidogrel, warfarin. *Cross Specialty medications include analgesics and immunosuppressants prescribed by multiple different specialties for multiple different organ indications. ADR, adverse drug reaction; PGx, pharmacogenomics.

Organ based speciality prescribers for PGx mitigatable ADRs are shown in Fig 3 (S6 Table). Over 47% of all the PGx mitigatable ADRs identified were attributed to psychiatric medications (*n* = 54,846). This was followed by cardiovascular medications, accounting for 24% (*n* = 28,279).

**Fig 4:**
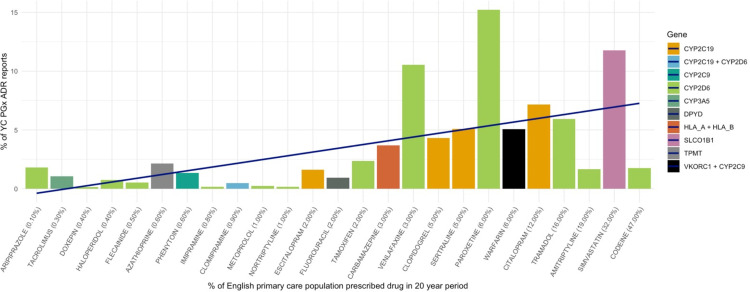
Proportion of ADRs per drug compared with prescribing prevalence. The *x*-axis denotes prescribing prevalence (% of the population prescribed the drug in a 20-year period (1993–2012) based on the published CPRD data from Kimpton and colleagues [16]). The *y*-axis shows the percentage of all PGx mitigatable ADRs represented by each drug during the same 20-year period. Those medicines which extend over the blue line are overrepresented in Yellow Card ADR volume as compared with prescribing prevalence. ADR, adverse drug reaction.

A comparison of prescribing prevalence from CPRD data with proportion of mitigatable ADRs associated with PGx drugs is shown in Fig 4 (S7 Table). It is notable that the majority of the PGx drugs with disproportionately high Yellow Card ADR reporting as compared with prescribing prevalence are psychiatric drugs.

The analysis of Oestrogen associated DVT and PE reports, which may be mitigated by avoiding these drugs in *F5* variant carriers, revealed that 3,917 DVTs or PEs were reported in association with use of different oestrogens. Fifty-two percent of these were DVTs and 48% were PEs. Most (73%) of these VTE reports were associated with ethinylestradiol and all of the reported VTE events were classed as either serious (86%) or fatal events (14%) (S8 Table).

The analysis of antidepressant- and antipsychotic-associated arrhythmia ADRs from PGx modifiable drugs revealed only 3% of the ADRs were cardiac arrhythmias (*n* = 1515), and fatal cardiac arrhythmias were uncommon consequences, comprising only 0.4% of these ADRs (*n* = 172 of *n* = 48,315 total ADRs). Of these cardiac arrhythmias ADRs 88% were severe and 11% were fatal, accounting for 10% of all fatalities associated with these drugs (S9 Table). Most of the antidepressant- and antipsychotic-associated ADRs from PGx modifiable drugs were severe or fatal (67% severe, 3% fatal) and are described in S10 Table.

The more extensive CPIC clinically actionable drug list (level A and associated with ADR risk) (64 drugs, S11 Table) suggests that 10.9% of ADRs may be mitigated (as compared with the 9% we found for the more stringent list validated by prospective clinical trial evidence).

## Discussion

**Fig 5:**
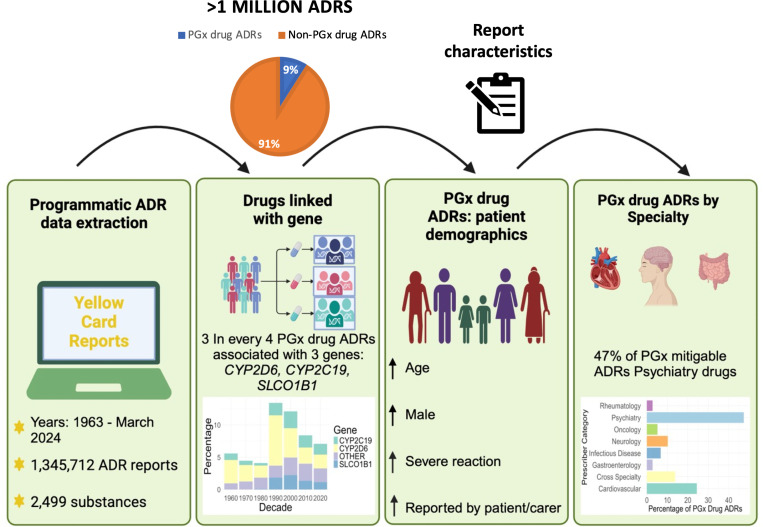
Study methods and results overview. PGx, pharmacogenomics; ADR, adverse drug reaction.

We have reported a national pharmacovigilance programme-based analysis of PGx drug-associated ADR reports at scale (Fig 5). We found that, among a large cohort of an excess of a million spontaneously reported ADRs during a span of 60 years, 9% were potentially mitigable by a pharmacogenomic approach. The prevalence of PGx mitigatable ADRs associated with these drugs has varied substantially by decade with peaks in reporting for single agents, most notably paroxetine, which contributed to a peak in PGx mitigatable ADRs in the 1990s.

Kimpton and colleagues found that drug interactions with only three pharmacogenes (*CYP2D6*, *CYP2C19* and *SLCO1B1*) account for the vast majority of PGx interactions in primary care prescribing (>95%) in an English setting [16]. Importantly, all of these genes impact on drug safety via pharmacokinetic pathways impacting the metabolism and excretion of the associated medications. Here, we find that those same limited three pharmacokinetic genes could potentially account for three in every four PGx mitigatable ADRs represented by the Yellow Card reports in the last five decades. This can inform pilot PGx implementation in direct healthcare by prioritising these genes. It is important to note that these are polymorphic genes that impact on the metabolism of many medications. This study characterised the most conservative estimate of PGx mitigatable harms, but we have also extrapolated the potential mitigatable harms if extended pharmacogenomic panels are proven prospectively to mitigate ADRs. As defined by the CPIC implementable list of drug–gene pairs this may extend the PGx mitigatable ADRs to 11%, an increment of 2% higher than our estimates presented here.

While we excluded oestrogens associated with increased risk of VTE in FVL from our PGx mitigatable group to give a conservative estimate, it is informative to see that nearly 4,000 VTE events have been reported, mostly associated with one formulation of oestrogen, ethinylestradiol. While it is unclear how many of these events may have been prevented using prospective PGx, 100% of these were serious or fatal, thus giving a powerful impetus to trial PGx implementation.

Trial data shows that of these potentially PGx mitigatable ADRs we might expect a 30% reduction with the use of prospective genotyping to inform prescribing [11]. If we extrapolate to Yellow Card data, then we can expect 34,737 reported ADRs may have been avoided with the limited 12-gene pharmacogenomic panel employed by the PREPARE trial [11]. The fact that the PGx mitigatable ADRs were more likely than non-PGx mitigatable ADRs to be severe has important healthcare cost implications. However, the PREPARE trial recruited based on indication for an index drug, included only 12 weeks of follow up, and actively asked participants about potential ADRs then adjudicated reactions to determine likely causal relationship with the medication. Therefore, this extrapolation is interesting, but may not be representative of the impact real world PGx prescribing would have on Yellow Card reporting of ADRs prospectively.

CYP2D6 and CYP2C19 are known to show large differences in prevalence of predicted extreme metabolizer types (poor metabolizers or ultrarapid metabolizers) across diverse ancestry groups due to varying prevalence of common genetic variants [17–19]. It is unfortunate that ancestry data was not available for these Yellow Card reports; however, based on what is known about polymorphism frequencies in these genes, that ADRs associated with a typical metabolizer types of associated drugs will be more frequent in under-represented African and Asian ancestry groups (Table 2) [2,17,18]. Therefore, piloting PGx with these few selective genes would not only capture 75% of all mitigatable ADRs but would also disproportionately benefit these populations therefore targeting existing health inequalities.

**Table 2:** A typical metaboliser prevalence for CYP2D6 and CYP2C19 across diverse ancestry groups. These are genetically predicted based on data published by PharmGKB and CPIC. We have defined atypical metabolisers as the following: poor (activity score 0 for 2D6), intermediate (activity score <1 for 2D6), and ultrarapid metabolisers (activity score >2 for 2D6).

Biogeographical groups	CYP2D6 atypical metabolisers	CYP2C19 atypical metabolisers
**African American/Afro-Caribbean**	22%	40%
**American**	10%	24%
**Central/South Asian**	15%	52%
**East Asian**	34%	59%
**European**	19%	33%
**Latino**	14%	23%
**Near Eastern**	28%	29%
**Oceanian**	22%	94%
**Sub-Saharan African**	25%	37%

Our analysis of national pharmacovigilance reporting at scale shows that the greatest potential area of intervention by specialty to mitigate Yellow Card reported ADRs in the UK may be within psychiatry. Psychiatric medication associated ADRs were the highest prevalence across organ systems (accounting for roughly half of all PGx mitigatable ADRs) and among the most overrepresented ADRs with respect to prescribing prevalence. This is synergistic evidence with the recently published secondary data from the PREPARE trial, which showed that those psychiatric patients with an actionable genotype in the intervention arm had a 34% reduction in ADRs, 41% reduction in hospitalisations and 41% lower readmission rates in addition to shorter hospital stays [20]. Although PGx mitigatable Yellow Card ADRs associated with psychiatric medication had a low prevalence of arrhythmias there were a substantial number of events reported, 1515, and 99% of these were either severe or fatal. Yet, arrhythmias represented only 10% of fatal ADR reports associated with these agents.

There are some limitations to the data presented. The voluntary nature of Yellow Card ADR reporting is likely to lead to potential biases and broadly acknowledged under-reporting. Milder and more predictable ADRs may be less likely to be spontaneously reported to the MHRA compared to atypical and more severe ADRs. The quality of the data may vary, with some reports containing incomplete information, and these reports have not been independently adjudicated. As catalogued in Table 1, there is a high level of missingness in some of the demographic data such as age. Age is supplied by the MHRA in this data rounded to the lowest decade as described in the methods section. Furthermore, information on medical comorbidities and other drugs taken at the time of the ADR were not available. However, the scale and duration make this dataset the most comprehensive nationally. Therefore, it is a valuable resource to examine the scope of potentially PGx mitigatable reported ADRs. Pharmacovigilance programmes, such as Yellow Card reporting, VigiBase and the FDA’s FAERS continue to be used because they have proven value in detecting safety signals despite limitations in the completeness and quality of ADR reports submitted.

Although the CPRD prescribing may not be representative of prescribing patterns in different subgroups it is likely that any bias would be toward healthier groups who engage in research. For example, the percentage of British South Asian participants from the Genes & Health cohort prescribed the antidepressants investigated is known to be higher than the CPRD data shows [21]. Therefore, the CRPD data would seem to provide a conservative and robust population level low-end estimate.

In conclusion, Yellow Card reports from the UK’s pharmacovigilance programme depict a national experience of reported ADRs since 1963. Our findings suggest that a limited panel of three pharmacogenes could potentially pre-emptively identify most of those at risk of ADRs and avoid significant patient harm and hospitalisation. Based on our findings, Psychiatry may be the single highest impact specialty to pilot PGx in the UK to reduce ADRs and associated morbidity, mortality and costs. These findings are based on the analysis of the Yellow Card dataset described and may not represent all ADRs from a generalised patient population.

## Supporting information

S1 Table List of pharmacogenomic drugs, their associated gene(s) and prescriber category. (XLSX)

S2 Table Number and percentage of adverse drug reactions associated with pharmacogenomic drugs stratified by decade. (XLSX)

S3 Table Number and percentage of adverse drug reaction reports associated with PREPARE drugs that have a pharmacogneomic indication for the gene SLCO1B1 stratified by decade. (XLSX)

S4 Table Number and percentage of adverse drug reaction reports associated with PREPARE drugs that have a pharmacogneomic indication for the gene CYP2D6 stratified by decade. (XLSX)

S5 Table Number and percentage of adverse drug reaction reports associated with PREPARE drugs that have a pharmacogneomic indication for the gene CYP2C19 stratified by decade. (XLSX)

S6 Table Number and percenateg of PGx assoicated adverse drug reactions stratified by prescriber category. (XLSX)

S7 Table PGx (number and percentage) associated adverse drug reactions and the prescribing prevalence (number and percentage) over a 20-year period (1993–2013). (XLSX)

S8 Table Number and percentage of pharmacogenomic mitigatable adverse drug reactions of oestrogen drugs. (XLSX)

S9 Table Psychiatric pharmacogenomic drugs stratified by type of adverse drug reaction (cardiac arrhythmia or other) and the level of seriousness. (XLSX)

S10 Table Psychiatric pharmacogenomic adverse drug reactions stratified by level of seriousness. (XLSX)

S11 Table Clinical Pharmacogenetics Implementation Consortium list of clinically actionable drugs with a pharmacogenomic indication and their associated genes. (XLSX)

S11 STROBE checklist. (DOCX)

## Abbreviations

ADRs: adverse drug reactions
CPIC: Clinical Pharmacogenetics Implementation Consortium
CPRD: Clinical Practice Research Datalink
DPWG: Dutch Pharmacogenetics Working Group
DVT: deep venous thrombosis
FVL: factor V Leiden
iDAPs: interactive drug analysis profiles
MHRA: Medicines and Healthcare products Regulatory Agency
PE: pulmonary embolism
PGx: pharmacogenomics
SSRIs: serotonin reuptake inhibitors
STROBE: Strengthening the Reporting of Observational Studies in Epidemiology
VTE: venous thromboembolism
